# Exploring member trust in German community-supported agriculture: a multiple regression analysis

**DOI:** 10.1007/s10460-022-10386-3

**Published:** 2022-11-07

**Authors:** Felix Zoll, Caitlin K. Kirby, Kathrin Specht, Rosemarie Siebert

**Affiliations:** 1 Leibniz Centre for Agricultural Landscape Research (ZALF), Eberswalder Str. 84, 15374 Müncheberg, Germany; 2 Michigan State University, 354 Farm Ln, East Lansing, MI 48824 USA; 3 ILS – Research Institute for Regional and Urban Development, Brüderweg 22–24, 44135 Dortmund, Germany

**Keywords:** Short food supply chains, Local food, Alternative food networks, Consumer trust, Trustworthiness, Food system transformation

## Abstract

Opaque value chains as well as environmental, ethical and health issues and food scandals are decreasing consumer trust in conventional agriculture and the dominant food system. As a result, critical consumers are increasingly turning to community-supported agriculture (CSA) to reconnect with producers and food. CSA is often perceived as a more sustainable, localized mode of food production, providing transparent production or social interaction between consumers and producers. This enables consumers to observe where their food is coming from, which means CSA is considered suitable for building trust in food (production). However, it remains unclear how exactly members’ trust in ‘their’ farmers is built. To determine the factors that predict members’ trust in CSA and its farmers, and the importance of these factors when compared to each other, we conducted a quantitative study among CSA members in Germany and applied a multiple regression model (*n* = 790). The analysis revealed that trust in CSA and its farmers is influenced by “reputation”, “supply of information”, “direct social interaction” and the “duration of CSA membership”. Other factors such as the “certification status of the CSA farm” and “attitudes toward organic certification” did not significantly predict trust. We conclude that producers’ willingness to be transparent already signals trustworthiness to CSA members and is more important to members than formal signals. Other actors within the food system could learn from CSA principles and foster a transition toward a more regionalized value-based food system to help restore agriculture’s integrity.

**Supplementary Information:**

The online version contains supplementary material available at 10.1007/s10460-022-10386-3.

## Introduction

Today’s globalized food system has become increasingly complex. Due to long, opaque food chains, food is handled, distributed and processed by countless actors before it ends up on people’s plates. Consumers can rarely discern exactly where, how and by whom their food is produced (Meyer et al. 2012; Thorsøe and Kjeldsen 2016). The growing spatial and social distance between producers and consumers creates a situation of uncertainty in which trust is necessary for consumers to make their food choices (Giampietri et al. 2018). The shared understanding of trust is that it is “a psychological state comprising the intention to accept vulnerability based upon positive expectations of the intentions or behavior of another” (Rousseau et al. 1998, p. 395). It means trust encompasses the assumption that in a situation of uncertainty, a person or institution will act appropriately. This creates an interdependence between actors, in which the interests of one party can only be achieved in reliance on each other. Consequently, trust is enhanced when the expected behavior actually occurs (Rousseau et al. 1998). However, these expectations are often not met. In the last decades trust in the food system and agriculture has been undermined for manifold reasons and at different stages of the food chain. Zoonoses such as bovine spongiform encephalitis (‘mad cow disease’), foodborne outbreaks of *E.coli*, food contamination with dioxin (Thorsøe and Kjeldsen 2016; Sage et al. 2020) or melamine, as well as incidents such as the horse meat scandal repeatedly raise concerns of food safety and question the authenticity of food and those who are involved in its provision (Manning and Smith 2015). Scholars frequently mention food scares as one reason for distrust in the current food system and consumers’ urge for different food production methods (Blay-Palmer 2008; Thorsøe and Kjeldsen 2016; Martindale 2021; Nemes et al. 2021). Furthermore, an increasing awareness of the negative environmental impact of agriculture on biodiversity add to a climate of distrust (Bos and Owen 2016; Busse et al. 2021). The recent COVID-19 pandemic exposed poor working conditions for seasonal field workers and slaughterhouse workers and resulted in a public outcry and additional criticism of the dominant system (Aday and Aday 2020; Friedrich et al. 2021). Even though lockdown restrictions also affected local farmers in terms of interactions with their customers and workforce shortages (Benedek et al. 2021) and a (complete) system failure in terms of food provision did not occur due to the COVID-19 pandemic, many people became more aware of grievances within the existing food system and the consequences of their food choices (Schoen et al. 2021; Vittuari et al. 2021).

Each of these developments contributed to the emergence and increasing popularity of alternative food production and distribution methods (Ostrom 2007; McGreevy and Akitsu 2016; Thorsøe and Kjeldsen 2016; Blättel-Mink et al. 2017; Schmidt et al. 2020; Nemes et al. 2021; Zollet et al. 2021). Alternative food networks are characterized by short supply chains, which supposedly allow direct interaction between producers and consumers, as well as social and economic community-building, and can restore transparent food production (Renting et al. 2003; Forssell and Lankoski 2015; Poças Ribeiro et al. 2021). Alternative food provisioning can encompass self-supply, models like community-supported agriculture (CSA), direct sales on farmers’ markets but also e-stores, where third parties establish cooperation with farmers to sell safe local food (Cicatiello 2020). What exactly builds consumer trust in food remains an unsolved yet central question for understanding increasingly popular alternative food networks (AFN). Therefore, this article aims to shed light on the trust-building factors in CSA.

In general, building a trusting relationship is highly complex, as the process depends on personal, social but also cultural factors (McGreevy and Akitsu 2016) and according to the literature, there are different measures for creating trust. Direct social exchange in the form of face-to-face interaction is commonly considered an important factor for trust development. However, relevant information can also be exchanged between actors in non-face-to-face situations (i.e. electronically) but the amount of information needed to create a certain level of trust (in such cases) is higher. A different form of proximity can also be created through reputation (Nilsson 2019). Reputation is a valuable form of self-presentation, created through informal mechanisms (Thorsøe and Kjeldsen 2016) and reliant on customer satisfaction (Walsh et al., 2009). I It raises expectations about future behavior and conversely, a person or organization who has managed to build a positive reputation has an incentive to behave in a way that maintains it (Thorsøe and Kjeldsen 2016). When direct contact between actors is not feasible, it is also possible to signal trustworthiness through institutional approaches established by third parties such as governments or private companies that regulate and safeguard the compliance with certain standards (i.e. organic certification) (Zhang et al. 2016).

While the mainstream food system seems to struggle in sufficiently addressing these measures for building trust, they play a major role in AFNs such as CSA. A high level of spatial and social proximity between producers and consumers is a key aspect of the AFN concept. In German CSA farms, a group of consumers typically agrees to pay a certain amount of money per month to a farmer over the course of a year and receives a share of the harvest in return, meaning that both parties share the risks and benefits of food production. The defining characteristics of CSA are their different forms of interaction between farmers and consumers, who are usually referred to as members (Opitz et al. 2019). Farmers try to directly address the needs of their members by establishing transparency and enabling their involvement, which can range from visiting the farm to helping with practical work in the field to sometimes even granting co-determination rights regarding farm decisions (Zoll et al. 2021). German CSA farms also usually apply organic production methods, although they are not necessarily certified (Schlicht et al. 2012; Kraiß and Meißner 2016). Furthermore, access to healthy food and the support of ecologically sound and socially just farming practices are a frequent motivation for consumers to join CSA (Cooley and Lass 1998; Zoll et al. 2018), indicating that positive expectations about CSA farmers’ behaviors do exist.

The previous focus of scholarly literature has been on exploring consumer trust in certificates or food safety, often from a marketing perspective (de Jonge et al. 2004, 2008; Hartmann et al. 2015), neglecting direct producer–consumer relations. However, Hendricksen and Heffernan (2002) point out that people do not place trust in food itself but rather in the producer to produce food safely. Filling this research gap is highly relevant, as the findings could possibly be transferred to rebuilding trust in agriculture and food systems. Following this argumentation, a growing body of qualitative studies from all over the world meanwhile argues that the described CSA setup has the capacity to create trusting relationships between farmers and members (Hinrichs 2000; McGreevy and Akitsu 2016; O’Kane 2016; Si 2017; Zoll et al. 2021). Consequently, CSA members do not have to rely on abstract regulatory measures to get assurance about food quality and farming practices (Feagan and Henderson 2009). In contrast, Pole and Gray (2013) state that this is only applicable in the case of an ideal CSA and raise the question as to whether these relationships actually exist. No study has been done that examines these contrary findings by quantitatively determining trust-building factors in CSA and quantifying the different impacts that the single factors have on trust when compared to each other.

Therefore, we address the following research questions:

1. Which factors influence the trust of CSA members in CSA and its farmers?
2. How strong is the impact of those factors on the trust of CSA members in CSA and its farmers?

## Development of the conceptual model

**Fig. 1:**
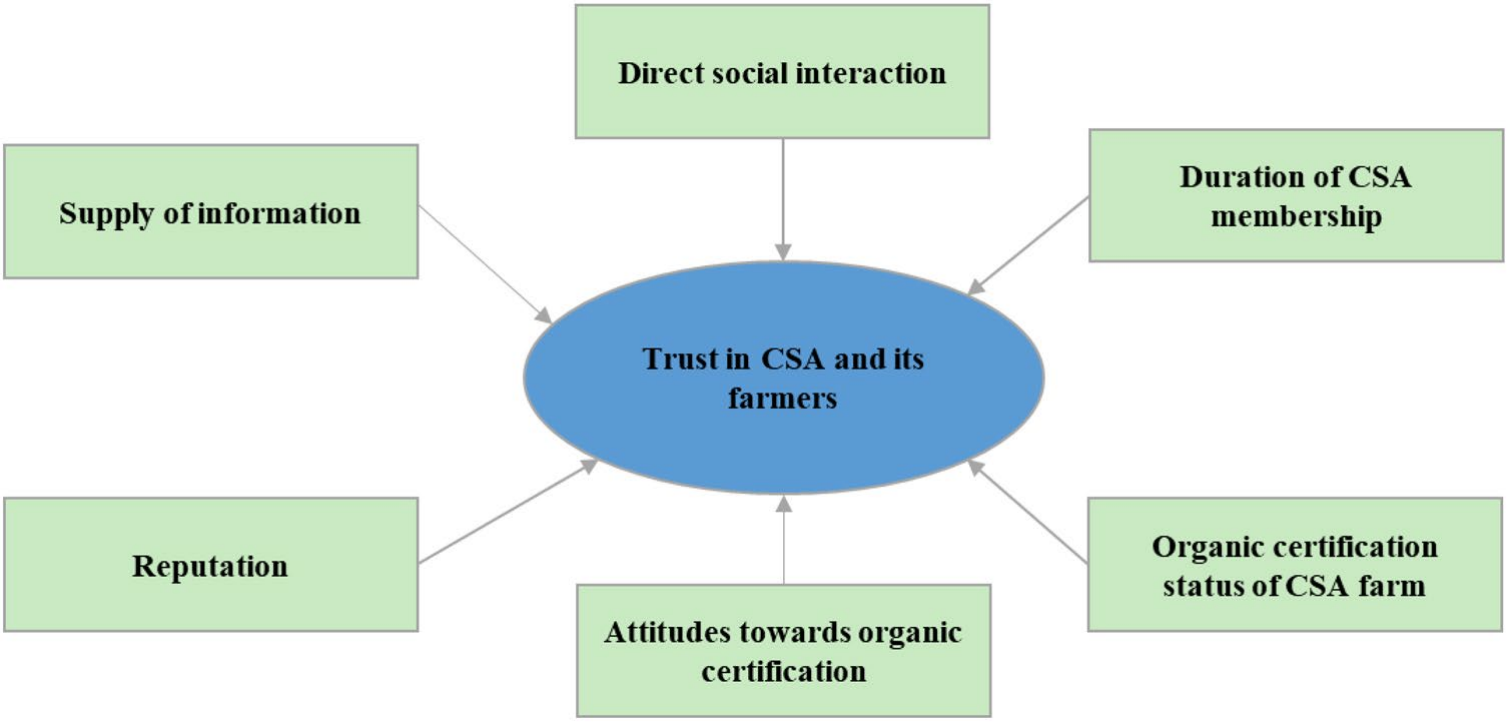
Graphic representation of the conceptual model (elements of the conceptual model correspond with the variables used in the regression model)

The conceptual model was derived from trust theory literature but also from studies on the relationship between trust and food-related topics in general, as well as findings from CSA and AFN literature. The following overarching topics were identified as possibly influencing trust in CSA and its farmers (see Fig. 1): “direct social interaction”, “supply of information”, “reputation”, “attitudes toward organic certification”, “organic certification status of the CSA farm”, “duration of CSA membership”, complemented with “attitudes toward short food chains” due to the latest developments following the COVID-19 outbreak.

### Trust in CSA and its farmer

Food from CSA is distributed directly to the members, meaning there are no intermediates or retailers involved (Koretskaya and Feola 2020). The shortened food chain puts the role of CSA and its farmers in the spotlight, as they are solely responsible for the quality and safety of the food, but consumers typically have more trust in farmers than in the three other main food system actors – government, manufacturers, and retailers (Ding et al. 2015). Approaches which enable the direct involvement of consumers are generally more trusted with regard to food safety (Zhang et al. 2016) and as long as CSA communities are relatively small, they seem suitable when it comes to creating personal trust (Thorsøe and Kjeldsen 2016). Consumer trust has to be invested in the competence of the farmer to produce food that is not harmful to consumers ‘health or the environment (de Jonge et al. 2008) and that the prices of a CSA membership accurately reflect the cost of producing accordingly, but also that fair working conditions prevail at the farm (Koretskaya and Feola 2020). We do not derive a hypothesis from these findings, as the aim of the article is to identify factors that influence trust in CSA and its farmer, which makes this our dependent variable.

### Direct social interaction

Several studies related to food and agriculture point to the importance of direct social interaction for trust building (Kneafsey et al. 2013; Zoll et al. 2018). On the one hand, face-to-face interaction enables the farmer to demonstrate that they are motivated to fulfil the members’ needs (Thorsøe and Kjeldsen 2016) by providing them with required information on food quality or environmentally responsible growing methods. At the same time, farmers can create an understanding of seasonality and the difficulties of food production among consumers (O’Kane and Wijaya 2015). Thus, producers are considerably reducing the complexity and uncertainty faced by consumers when it comes to food procurement (Giampietri et al. 2018). Direct interactions also make it possible for the farmer to display their authenticity and passion, as well as the meaning they derive from food production and the intrinsic value of food, which goes far beyond aesthetics (O’Kane and Wijaya 2015). Furthermore, these interactions can create a feeling of appreciation for producers (Sage 2003). Continuously meeting the same person not only fosters personal trust in the farmer but also systemic trust (Thorsøe and Kjeldsen 2016). In our case, systemic trust would be expressed as trust in CSA farms as an organization.

In CSA there is also direct social interaction taking place among the members, for example when they work together during harvest or meet at farm events (Zoll et al. 2021). Other studies found that other peoples ‘subjective norms influence a person’s intentions when it comes to performing a behavior related to food (Giampietri et al. 2018; Carfora et al. 2019), which suggests that, depending on the intensity, member interaction could influence the way somebody feels about CSA. In CSA, direct social interactions have, for instance, contributed to a sense of community and belonging (Zoll et al. 2018). Following these findings, we propose the first hypothesis:

#### H1

Direct social interaction influences trust.

### Supply of information

If a food network encompasses a large amount of people, it is almost impossible for a producer to establish and maintain direct interactions with all the members, but there are other ways of communication which reduce the information asymmetry and create transparency: Farmers can, for example, use a website or social media to present their farms (Thorsøe and Kjeldsen 2016). Online communication has become increasingly popular for facilitating continuous member contact, which is regarded as crucial to create trust. It is used to satisfy member interests, create awareness and educate members about the material qualities of food, which is an important aspect, as members do not always perceive the material qualities positively (Martindale 2021). Among German CSA farms, it is quite common for members to be regularly sent a newsletter that informs them about the latest developments at the farm and in the field (Zoll et al. 2021). The information exchange has not only proven to foster AFN participants’ learning about production processes but also their understanding of farmers’ perspectives (Opitz et al. 2017). Farmers demonstrating openness is furthermore considered an important factor in creating trust (Macready et al. 2020) which leads us to the second hypothesis:

#### H2

Supply of information influences trust.

### Reputation

Reputation is a complex construct that describes peoples’ perceptions and beliefs of an entity (e.g. an organization or a company) (Walsh et al. 2009; Rindova et al. 2010). It can be an intangible asset for a business if it is evaluated positively by its stakeholders (Rindova et al. 2010). In this respect, reputation as a form of self-presentation also defines what others expect from a person or business (Thorsøe and Kjeldsen 2016). In food-related studies, reputation is described as a trust-creating informal signal, as it depicts a social control mechanism which motivates food actors to act reliably according to consumers’ expectations, thus avoiding negative feedback and maintaining a positive reputation (Thorsøe and Kjeldsen 2016; Tonkin et al. 2016). When considering current customers of a business, customer satisfaction is a key antecedent to reputation (Walsh et al. 2009). In regard to CSA, Martindale (2021) demonstrated that reputation plays an important role because members often use their personal networks to attract new members by passing on their experiences with CSA and the farmer to a third party. Generally, word-of-mouth between customers can be more influential on a company’s reputation than traditional marketing (Walsh et al. 2009). Characteristics of a CSA that build reputation therefore include customer satisfaction, expectation of a CSA’s future behaviors, and willingness to recommend a CSA to others. Thus, we hypothesize:

#### H3

Reputation influences trust.

### Duration of CSA membership

It is not only the form of interaction that contributes to trust-building but also the frequency and duration of interactions. Relationships over a longer period of time, which enable reoccurring contact, create stronger trusting ties between producers and consumers (McGreevy and Akitsu 2016; Thorsøe and Kjeldsen 2016). In accordance with these assumptions, we suggest:

#### H4

The duration of the CSA membership influences trust.

### Organic certification status of the CSA farm

Guthman (2004) proposed that organic certification provides a valuable signal for consumers, as they attribute qualities to certified food as being preferable to conventionally produced food. CSA members can usually visit their farm in person to obtain information on production methods, but this way is time-consuming so members might weigh effort against the utility of being able to personally verify information regarding the origin of their food or to rely on a label (Peterson et al. 2015). Pole and Kumar (2015) confirmed that certain groups of members joined CSA because they particularly value local, fresh organic food and concluded it might be in farmers’ interest to appeal to them by undergoing organic certification. This leads us to the proposition:

#### H5

The organic certification status of a CSA farm influences trust.

### Attitudes toward organic certification

The presence of a label does not necessarily create trust, this also depends on consumers’ attitudes toward certification (Janssen and Hamm 2014). CSA consumers typically express considerable concern about the ecological footprint of their food consumption, and oppose the use of pesticides or factory farming (Zoll et al. 2018). If food attributes cannot be observed directly by consumers, organic labels can provide information about food safety or production methods. Organic certification is therefore a frequently mentioned market-based regulatory approach that is supposed to create trust in food (Zhang et al. 2016). A food label can act as a surrogate for direct interaction with food producers (Tonkin et al. 2016) which means that consumers do not have to negotiate production standards with their farmer themselves, as these are institutionally monitored (Thorsøe and Kjeldsen 2016). However, the effectiveness of certification is also based on whether it is perceived as credible (Ward et al. 2004). Based on these results, we derive the following hypothesis:

#### H6

Attitudes toward organic certification influence trust.

### Socio-demographic factors

We refrained from including socio-demographic factors such as gender, education or income in the conceptual model following Macready et al. (2020) who did not consider them in their study on trust in food actors, as the patterns of effects in other studies were not consistent. Similarly, Peterson et al. (2015) state that demographic variables are not likely to properly predict behavior regarding CSA participation. There are food-related studies dealing with trust and socio-demographic factors, but their context does not align with our study. For example, Wu et al. (2013) found that higher levels of education among consumers go hand-in-hand with lower willingness to purchase food with additives, even if this complies with government regulations. Another study by Galt et al. (2017) explores differences in CSA membership experiences for low- and high-income members and found that the largest difference between the two groups was the perceived importance of affordability. Low-income members found it more important to personally know the farmer, but this was statistically insignificant.

## Research design and methods

### Research design

We designed a cross-sectional study of German CSA members using convenience sampling. We chose to use a survey as our data collection instrument in order to get data from a large number of CSA participants, and contribute a quantitative study to the literature on trust in CSAs. Based on our conceptual model, we developed a survey to be able to empirically test the hypotheses introduced in the previous section.

### Data collection

In total, the survey consisted of 69 questions which were mainly closed (see Annex 1 in supplementary material for the entire survey). However, not all questions have been included in the data analysis. A major part of the survey was dedicated to trust (seven items) and the hypothesized trust-building constructs of “direct social interaction” (eleven items), “supply of information” (seven items), “reputation” (eight items) and “attitudes toward organic certification” (seven items). We measured each of the constructs using several items that respondents could rate on a five-point Likert-scale, ranging from ‘fully agree’ (5), ‘rather agree’ (4), ‘neither agree nor disagree’ (3), ‘rather disagree’ (2), ‘fully disagree’ (1). We also used the data on the “duration of respondents’ CSA membership” (one question) and the “organic certification status of the CSA farm” (one question) for hypothesis formulation. Furthermore, the survey captured respondents’ socio-demographic information, of which we used gender, age and place of residence (three questions).

An online version was then set up using SoSci-Survey software (Leiner 2019). This version was pre-tested among colleagues and several CSA members to ensure technical functionality as well as scientific and thematic quality, and adapted according to the feedback. Sharing the survey with CSA members entailed several obstacles: There is no sampling frame of CSA members and data protection laws in Germany prevent the farms from sharing contact information of their members. Whenever no sampling frame of a population of interest is available, like in our case, non-probability sampling has to be applied (Galloway 2005). In the context of food production and consumption this has for example been done to explore groups such as farmers who conduct direct marketing (Azima and Mundler 2022) or consumers of local food (Skallerud and Wien 2019). We used Pole and Kumar`s (2015) study who also researched CSA members as an orientation for our sampling. We personally contacted all CSA farms who were organized within the German network for community-supported agriculture (Netzwerk Solidarische Landwirtschaft) at that time and were available via email. Overall, 267 farms were invited to forward the online survey to members. As CSA farms want to keep email traffic for their members low and the response rate was good, no reminders were sent. The survey was open to respondents from October 29, 2020 until January 15, 2021 resulting in 990 responses. Our approach resembles network sampling where initial ‘seeds’ that are part of the population of interest are asked to participate in the survey and then spread the survey among their contacts. This sampling method is considered as particularly useful when conducting research on small or hard to reach populations (Baker et al. 2013). Although drawing statistical inference from non-probability data to the entire population of interest has been proven to be possible (Fabo and Kahanec 2018), doing so is debatable. While the results of non-probability sampling cannot be called estimates, they can still be considered as indications as almost all non-probability samples contain a certain level of natural randomization (Baker et al. 2013). Therefore, we regard our study as rather explorative and do not claim that the results of the data analysis are representative for all CSA members in Germany.

### Data analysis

Data analysis was conducted using Stata version 16 (StataCorp 2019).

#### Factor analysis

We conducted exploratory factor analysis on each set of items for our dependent variable “trust” and independent variables “direct social interaction”, “supply of information”, “reputation”, and “attitudes toward organic certification” to ensure the validity of our scales (Knekta et al. 2019). We calculated Kaiser–Meyer–Olkin values and conducted Bartlett’s test of sphericity to determine that the data was appropriate for factor analysis (Bandalos and Finney 2018). Factors were extracted using the principal factor method, with the number of factors forced to one for each set of items. Items were removed iteratively based on low loadings (< 0.35). Factor loadings for items on each scale were all > 0.37 (see table S1 in supplementary material for detailed factor loadings) and Eigenvalues were all greater than one. The Cronbach’s alpha values for all of our factors was > 0.7, which indicates adequate internal consistency and reliability (OECD 2008). Following factor analysis, we averaged across each individual’s item responses in each scale to create a scale score ranging from 1–5. Averaging across several individual Likert items that have satisfactory factor analysis results transforms the data so that it can be used as continuous (DiStefano et al. 2021).

#### Multiple regression

We built a regression model following our conceptual model (see Fig. 1) and the results of the factor analysis (see table S1 in supplementary material). The dependent variable “trust in CSA and its farmers” is a scale consisting of seven items. Five out of seven independent variables are scales as well: “direct social interaction” (11 items), “supply of information” (seven items), “reputation” (eight items), and “attitudes toward organic certification” (seven items). Furthermore, two categorical variables – “organic certification status of the CSA farm” (no = 0/yes = 1) and “duration of CSA membership” (less than three years = 0/more than three years = 1) were included. Datasets with missing responses were removed prior analysis resulting in a sample size of n = 790.

To reveal the relationship between the dependent variable “trust in CSA and its farmers” and the independent variables, multiple regression analysis was conducted. An ordinary least squares (OLS) model was applied as its strength is to exhibit the influence of each independent variable on the dependent variable while controlling for the effects of the other variables (Galt 2013). All independent variables were entered into the regression simultaneously. Data was checked in advance to ensure it was appropriate for multiple regression analysis. Visual inspection of residual plots did not result in indication for violation of linearity or homoscedasticity. The variance inflation factors (VIF) are slightly bigger than 1 suggesting there are no multicollinearity issues. The Durbin-Watson-Test (1.973) indicates that the residuals are independent.

## Results

In this section, we start by reporting the characteristics of the sample that was used for regression analysis. We then provide insights into the results of the factor analysis and the variables on each scale. Finally, we proceed with the results of the multiple regression analysis.

### Sample description

The majority of respondents was female (70.4%) while the two biggest age groups consisted of the 30–44 (38.0%) and the 45–59 (27.1%) year-olds. In regard to the duration of CSA membership, 68.2% had been a member for less than three years and 31.7% for more than three years. More precisely, the biggest group (26.6%) joined their CSA farm less than one year ago. Among the farms, 54.1% did not have organic certification but applied organic farming practices, slightly less farms were certified (45.9%) (see Tables 1, 2).

**Table 1:** Characteristics of respondents (n = 790)

Gender	
Female	70.4%
Male	26.6%
Diverse	1.1%
No comment	1.9%
Age	
18–29	17.6%
30–44	38.0%
45–59	27.1%
60–65	8.5%
Above 65	8.0%
No comment	0.9%
Duration of CSA membership	
Less than one year	26.6%
≥ 1 year and < 2 years	24.7%
≥ 2 years and < 3 years	16.9%
≥ 3 years and < 4 years	11.5%
≥ 4 years and < 5 years	7.5%
Less than six years	11.8%
More than six years	0.9%

Percentages may not total to 100% due to rounding

**Table 2:** Organic certification status of the CSA farm (n = 790)

Not certified but applying organic principles	54.1%
Certified	45.9%

**Fig. 2:**
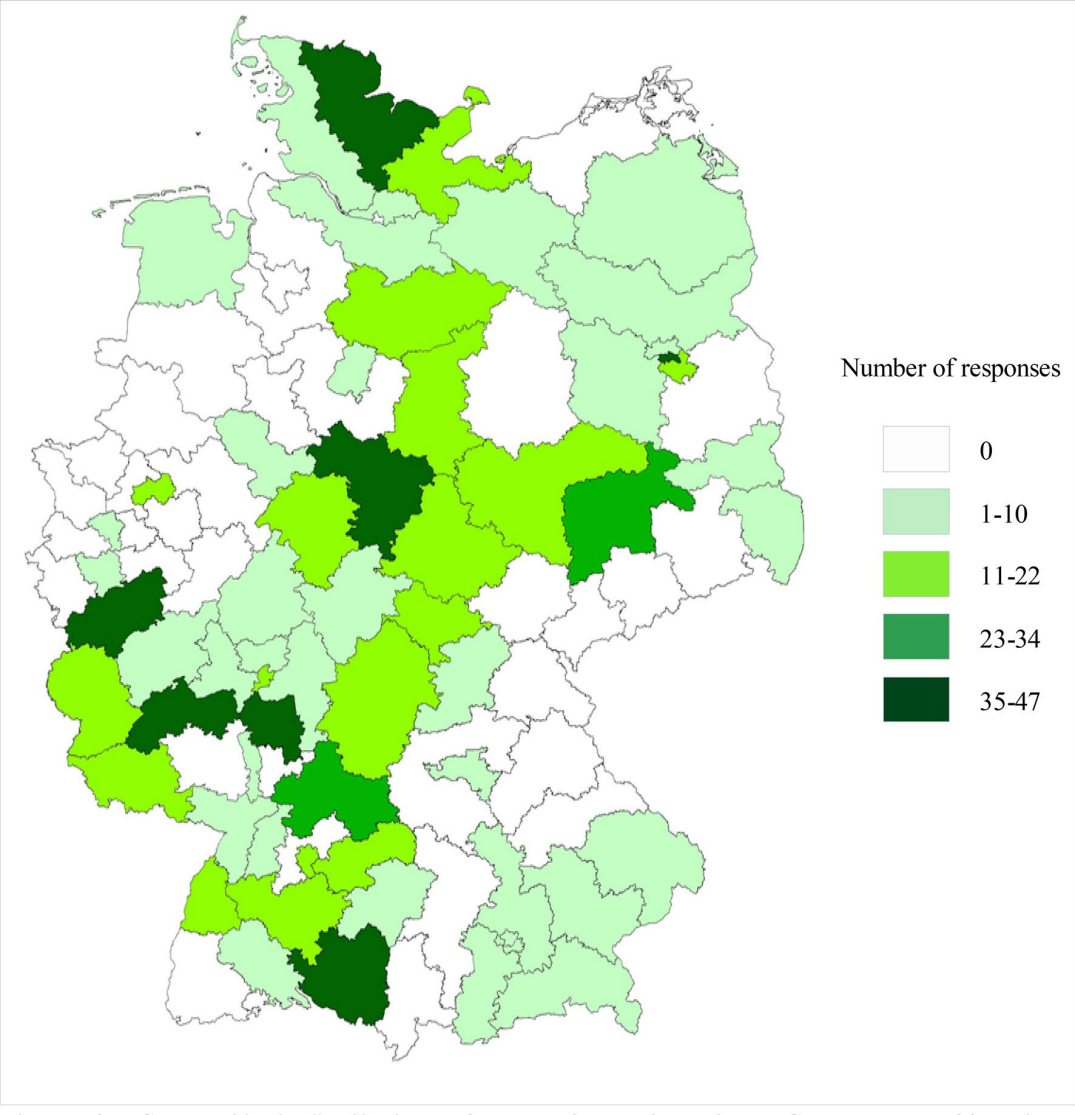
Geographical distribution of respondents throughout Germany, n = 790 (own representation based on OpenStreetMap data, © OpenStreetMap-Mitwirkende)

The graphical response distribution highlights that responses were captured from all over Germany, except some regions in the west and east. The majority of responses came from the center and South-western Germany (see Fig. 2).

### Overview on trust in CSA and its farmers and its potentially influencing factors

The results illustrate that trust in CSA and its farmers is high overall. All means of the individual items related to aspects such as effort to produce the best possible product quality, to produce according to environmental standards, general trust, food safety or labor conditions are also close to the maximum value of 5 (see Table 3).

**Table 3:** Trust in CSA and its farmers

Statement (1 = Don ‘t approve at all, 5 = fully approve)	Item scores (mean ± std. dev.)
I trust that my CSA tries to achieve the best possible product quality	4.86 ± 0.42
I trust that my CSA sticks to environmental standards	4.83 ± 0.40
I generally trust in my CSA	4.82 ± 0.41
I generally trust in the farmer(s) of my CSA	4.81 ± 0.49
I trust that my CSA does not overcharge for their products	4.77 ± 0.51
I trust that my CSA can guarantee for the safety of the food that they supply	4.69 ± 0.53
I trust that my CSA provides their employees with fair labor conditions	4.65 ± 0.65
Averaged scale score	4.78 ± 0.35

The results for the items constituting direct social interaction indicate that on average, members are satisfied with the degree of participation and the feedback they receive on products and production methods. The possibility of visiting the farm in person is of high importance and the possibility of interacting with farmers increases faith that they conduct agriculture in a responsible manner. In comparison, knowing the farmers’ motivation for growing food and particularly interactions with other members are less important to the respondents (see Table 4).

**Table 4:** Direct social interaction

Statement (1 = Don ‘t approve at all, 5 = fully approve)	Item scores (mean ± std. dev.)
In my CSA there are sufficient opportunities for member participation	4.64 ± 0.62
In my CSA I get sufficient feedback regarding products and production methods	4.53 ± 0.69
It is important to me to have the possibility to visit the CSA to personally get an idea of the production of my food	4.51 ± 0.76
The possibility to directly interact with farmers increases my faith that my CSA farmer(s) conduct agriculture responsibly	4.46 ± 0.75
It is important to me to be able to get direct feedback from farmer(s) in case I have questions regarding their products or production methods	4.10 ± 0.94
There is a sense of community between members and farmer(s) in my CSA	4.08 ± 0.88
The social interactions in my CSA are an enrichment for me	3.95 ± 1.07
It is important to me to have the opportunity to participate in my CSA	3.90 ± 1.00
In my CSA I have sufficient interactions with other members	3.85 ± 1.02
It is important to me to know the personal background of the farmer and to know what their motivation to grow food is	3.74 ± 1.01
Interactions with other CSA members are important to me	3.52 ± 1.04
Averaged scale score	4.11 ± 0.55

The high overall mean for supply of information indicates that the members are content with the information flow. Specifically, there is high approval for assuming that the CSA farms are providing correct information and making production, processing but also financial operations sufficiently transparent. Additionally, the respondents rated their satisfaction with the amount of information higher than the degree to which they are interested in the provided content (see Table 5).

**Table 5:** Supply of information

Statement (1 = Don ‘t approve at all, 5 = fully approve)	Item scores (mean ± std. dev.)
I assume that the information provided by my CSA are correct	4.77 ± 0.56
My CSA provides sufficient transparency regarding production and processing of their products	4.69 ± 0.61
Transparent production processes are generally important to me	4.58 ± 0.62
My CSA provides sufficient information on what the members’ financial contributions are used for	4.54 ± 0.77
I am satisfied with the amount of information that my CSA provides online	4.21 ± 0.90
Being a CSA member has increased my knowledge about food	4.11 ± 1.08
The content of information provided online by my CSA is interesting to me	3.96 ± 0.94
Averaged scale Sscore	4.41 ± 0.51

The reputation of CSA farmers is perceived as highly positive overall. Looking at the individual items comprising reputation, the members expressed high satisfaction concerning their experiences with the CSA, i.e. regarding the reliability of quality or delivering the harvest shares on time. Building on those experiences, the members’ expectations that the CSA farm will be reliable in the future is also high. The statements that members share the same values with the CSA farm regarding food and would recommend their CSA farm to peers were also highly approved by the respondents (see Table 6).

**Table 6:** Reputation

Statement (1 = Don ‘t approve at all, 5 = fully approve)	Item scores (mean ± std. dev.)
Due to my prior experience with my CSA, I think that my harvest share will be delivered / will be available to me reliably in the future	4.79 ± 0.48
My harvest share has been delivered / has been available to me reliably	4.74 ± 0.53
I would recommend to people who are important to me to join my CSA	4.72 ± 0.61
I am willing to accept flaws caused by my CSA because I think that the operators generally do their best to avoid mistakes	4.71 ± 0.55
Regarding food, my CSA and I share the same values	4.69 ± 0.57
Due to my prior experience with the CSA, I think that the CSA will also meet my expectations regarding the quality of the products in the future	4.65 ± 0.62
The quality of my CSA produce meets my expectations	4.58 ± 0.62
The price of the harvest share / products is appropriate	4.48 ± 0.72
Averaged scale score	4.67 ± 0.40

The overall mean of attitudes toward organic certification is lowest when compared to the other overall scale scores. Regarding the individual items, organic certification is generally an important shopping criterion for the CSA members, but the results also illustrate that people would like to have more information on the products than the organic label can provide. This is in line with the comparably low mean approval that organic certification ensures that farmers produce according to the required standard and the level of knowledge concerning which organic label entails which production standard (see Table 7).

**Table 7:** Attitudes toward organic certification

Statement (1 = Don ‘t approve at all, 5 = fully approve)	Item scores (mean ± std. dev.)
Organic certification is generally an important criterion to me when shopping groceries	4.10 ± 0.91
I know where I can get information on organic labels	4.10 ± 0.97
Organic certification gives me security that the product has attributes that I cannot observe	4.01 ± 0.91
When I buy organic food I pay attention which specific organic label the product has	3.71 ± 1.08
Organic certification ensures that farmers produce food according to the required standard	3.62 ± 0.82
I know the different standards that different organic labels entail	3.60 ± 1.02
If food has an organic label I do not need additional information on the product	2.69 ± 0.95
Averaged scale score	3.69 ± 0.62

### Multiple regression

The overall model was found to be significant (F(6, 783) = 97.72, p < 0.01), which means that the group of independent variables reliably predicts the dependent variable. Together, the predictors of our model explain 42.82% of the variance of trust in CSA (adj. R^2^ = 0.428).

The results of the OLS regression provide information on which factors influence trust in the CSA and its farmers and therefore answer our first research question. Reputation (p = 0.000) and Supply of information (p = 0.000) were highly significant predictors. Furthermore, the duration of CSA membership (p = 0.012) and direct social interaction (p = 0.003) were statistically significant in explaining trust in CSA and its farmers. We therefore accept H1, H2, H3 and H4. The organic certification status of the CSA farm (p = 0.092) and attitudes toward certification (p = 0.462) did not demonstrate a significant impact on trust in CSA and its farmers at a 0.05 level. This leads us to the rejection of H5 and H6 (see Table 8).

**Table 8:** Multiple regression predicting trust in CSA and its farmers

Predictor	β	p	Hypothesis	
Reputation	.488***	.000	3	Accept
Supply of information	.208***	.000	2	Accept
Direct social interaction	.0923*	.003	1	Accept
Duration of CSA membership	-.0699**	.012	4	Accept
Organic certification status of the CSA farm	-.046	.092	5	Reject
Attitudes toward organic certification	.021	.462	6	Reject

***Variable is significant at the 0.05 level

*Variable is significant at the 0.01 level

The regression model also indicates which independent variable has the strongest impact on trust creation, answering the second research question. The first column of Table 8 displays that reputation had the strongest impact on trust as it had the highest standardized regression weight by far (β = 0.488). The second most important factor affecting trust was the supply of information (β = 0.208). The beta weight for direct social interaction (β = 0.0923) was considerably lower, followed by the duration of CSA membership (β = -0.0699). The significant independent variables “reputation”, “supply of information” and “direct social interaction” are all positively related to trust in CSA and its farmers, meaning that a higher rating regarding these factors increases trust. “Duration of CSA membership” is an exception, being negatively related to trust, which suggests that people who have been a member for more than three years have slightly less trust in CSA and its farmers than people who have been a member for less than three years, holding all other variables constant.

## Discussion

In trust literature, ***direct social interaction*** is usually mentioned as the most important measure for creating trust (Nilsson 2019). According to our results, direct social interaction is only the third most important predictor of trust. This raises the question as to what kind of interaction the members desire. The high importance of having the possibility of interacting with the farmers and going to visit the farm indicates that the mere willingness of farmers to present their farms is already a credible signal of trustworthiness for members. Indeed, the actual participation in farm events and activities is rarely a motivation for people to join CSA, leading to farmers often farming alone (Pole and Gray 2013). Oberholtzer (2004) identified the most common reasons for CSA members’ absence in farm activities as being time constraints and scheduling conflicts. As CSA participation also comes with additional time requirements for picking up and cooking food (Zoll et al. 2021), our results support the assumption that less time-consuming forms of accessing information on their food are more important for member trust. In fact, in our study, reputation had the highest impact on trust-building, followed by supply of information.

The high importance of ***reputation*** in our study supports the assumption that consumers analyze producers’ behaviors, exchange the produced knowledge and align their consumption decisions accordingly (Randelli and Rocchi 2017). In our survey, reputation was mainly expressed through member satisfaction, which was high, and respondents also exhibited a high willingness to recommend their CSA farm to peers. As scholars such as Ward et al. (2004) note that reputation is difficult for farmers themselves to establish, the importance of word-of-mouth for the success of a CSA farm is underlined. Both members’ personal social networks (Martindale 2021) and the use of digital platforms can facilitate information dissemination and therefore reputation-building, but the internet in particular has turned reputation into a phenomenon of whose importance bridges society, as nowadays anyone can rate and compare anything online (Räwel 2020). Spreading reputation online, for example through positive reviews (Schor and Wengronowitz 2017), reduces the dependence of trust on spatial proximity (Bolton et al. 2013). Once a critical mass perceives someone’s reputation as positive, others will not only quickly develop trust about them as well, it is also less likely that a negative event will significantly reduce the trust in the individual (McKnight et al. 1998). For this indirect creation of social proximity, it is important that the farmer manages to present themselves as a part of the community they want to appeal to (Nilsson 2019). So even though reputation is second-hand information, possibly replacing direct interaction, activating third-party referral still requires skills and effort from farmers.

***Supply of information*** was the second most important predictor of trust in CSA and its farmers in our model. Macready et al. (2020) even identified demonstrating openness by conveying relevant information as most important leverage for creating consumer trust in food chain actors. The changing information communication landscape, in particular, offers different opportunities for creating transparency, as it makes food chain actors observable (Macready et al. 2020). In Japan, where the CSA principles emerged, various on- and offline means of supplying additional information exist. It is quite common, for example, for food to be sold together with photos and information about the farmer and their farming practices. By providing the consumer with a photo and their personal story, the farmers’ faces form an affective seal of quality (McGreevy and Akitsu 2016). Another innovative promising way of reconnecting producers and consumers stemming from Japan is interaction via smartphone apps. Consumers can sometimes even influence the growing plan and receive photos and status reports of the growing process (McGreevy and Rupprecht 2017). Bos and Owen (2016) also point to the importance of farmers providing information to consumers, for example through newsletters or social media, to increase authenticity, enable communication and a social reconnection without actually meeting in person. Conversely, they emphasize that those forms of virtual reconnection tend to supplement rather than replace offline social interaction when it comes to creating trust and transparency. Similarly, Ji et al. (2019) demonstrated that offline contact is an important first step before trust can be built online. Furthermore, trustworthiness can be created through transparency and the provision of information only if they are accompanied by the communication of values and how they are applied in respective actions (Meijboom et al. 2006). In any case, providing members with information through channels which they can access whenever they have time seems to be more flexible than participating in direct interactions.

***The duration of CSA membership*** also significantly affects trust, but to our surprise, the relationship was negative even though long-term members’ trust is only slightly lower when compared to short-term members. Following other literature on trust theory and trust in food, a longer relationship is often associated with an increase in trust (Brashear et al. 2003; McKitterick et al. 2019). However, trust can also evolve at different speeds depending on the person and how they evaluate a trustee at different stages of the relationship (Akrout and Diallo 2017). Furthermore, other research found that long-term relationships are possibly more prone to negative impacts (Moorman et al. 1992). In our case, it appears as though short-term members might still have a more romanticized image of agriculture and CSA. A more detailed look at our data on CSA membership reveals that more than 25% of all respondents joined their CSA within the last 6 months, i.e. during the Covid-19 pandemic. So, a possible explanation could be that people had developed a strong distrust in conventional food chains and therefore possibly had stronger trust in CSA at the moment they joined, but this explanation should be explored further.

In our results, neither the ***organic certification status of the CSA farms*** nor ***attitudes toward organic certification*** were a significant predictor of trust in CSA and its farmers. This represents a partial contrast to other studies, in which organic certification and the associated control system is mentioned as a measure for creating trust in food and food actors (Janssen and Hamm 2011; Zhang et al. 2016). However, a closer look into the literature reveals quite differentiated results: Bildtgard (2008) argues that food labels administered by NGOs in particular would transport certain community-based norms and values as well as political agendas and thus appeal to certain activist groups related to the environment, animal rights or other social movements. Due to the international character of food labels, he even regards them as a means to define the postmodern foodscape. In Germany, different consumer segments prefer different labels, i.e. frequent buyers of organic food prefer stricter labels. Trust in labels such as the EU logo is low, but food that is labelled as organic but does not display a certification logo is distrusted as well (Janssen and Hamm 2014). As German consumers using alternative forms of food supply are often in opposition to the conventional system (Zoll et al. 2021), it is likely that CSA members tend to be critical toward certification schemes controlled by mainstream institutions as well. Veldstra et al. (2014) found that farmers who both apply organic farming principles and distribute their products through direct marketing channels are less likely to certify their products. From a consumer perspective, there is similar evidence, i.e. consumers who shop at so called participatory farmers’ markets prefer the high level of interaction occurring there over organic certification (Moore 2006). Together with our results that the majority of our surveyed farms do not have organic certification, and the comparably low averaged scale score for attitudes toward organic certification, this suggests that CSA consumers preferably rely on their own experiences related to reputation, supply of information and direct social interaction. As a result, especially small farms that only use direct marketing channels such as CSA and do not sell to wholesalers could refrain from undergoing costly, bureaucratic and time-consuming certification processes (Veldstra et al. 2014; Kondoh 2015). Participatory guarantee systems (PGS) which are common in Latin America have already proven to successfully replace formal certification, mutually benefitting both consumers and producers. They are closely related to CSA principles as they are based on regenerative agriculture, and trust is created through different forms of consumer participation, shared values, and responsibilities (Marchetti et al. 2020). Forgoing organic certification does also not necessarily reduce the income that CSA farmers can generate with their produce. Guthman (2004) uses the term “community rent” to describe members’ willingness to pay more for the belief that farmers create transparency and trust, which symbolizes a commodification of the close relationship between members and producers.

Our Study faces several ***limitations***. As data on CSA members in Germany is limited with regard to aspects such as demography, total number of both CSA farms and members or contact information, probability sampling was not possible. Consequently, we depended on contact information of the German CSA network to spread the survey invitation among CSA farms asking them to forward it to their members. This, first of all, means that CSA farms outside the network were not contacted. Secondly, we were neither notified by all of the farms who forwarded the survey invitation, nor aware of the member number of the participating farms which also means that we could not calculate a response rate. It is furthermore possible that farms with a high number of members are overrepresented in the sample. Other studies on CSA members were limited by similar constraints (Pole and Kumar 2015). As a result of the non-probability sampling, our study may not be representative and cannot be generalized to our population of interest. There is a scholarly debate that random sampling does not automatically produce representative results (e.g. due to high non-response rates) and results based on non-random sampling are not inherently invalid, but have been proven to be equally reliable on several instances (Galloway 2005; Baker et al. 2013). Nevertheless, our exploratory results should be tested again, ideally using a dataset based on random sampling. ***Methodologically***, our regression model includes different factor scores based on Likert scales (DiStefano et al. 2021). In scholarly literature there has been a long debate on whether or not Likert scales can be used as factor scores (Carifio and Perla 2008). However, there is evidence to suggest that means obtained from Likert scales are perfectly adequate for inclusion in parametric tests such as multiple regression or ANOVA, yielding in unbiased results. This is particularly the case when, as in our case, the data is normally distributed and techniques such as Cronbach’s alpha and factor analysis are applied beforehand to ensure that the grouped items measure the underlying variable (Sullivan and Artino 2013).

In our study, we explored the importance of different trust-building factors when compared to each other, particularly highlighting the importance of reputation, supply of information and direct social interaction. However, the results also point to ***further research needs***: First, more in-depth research, for example using qualitative interviews, is needed to explore exactly which kind of information and interaction CSA members need in order to build trust. Second, trust is not only an outcome variable of certain activities that the CSA provides, but at the same time a necessary precondition for the willingness to participate in the CSA. In our survey, we did not include a way of measuring the “starting level” of trust, which must have been already existent when members joined the CSA. Third, possible positive and negative feedback loops between the factors were beyond the scope of our statistical analysis. Therefore, future research could consider if and how the factors we investigated influence and interact with each other. Last, our study also only focuses on CSA members, who might have different needs than other consumers. It would therefore be interesting to conduct a large-scale study that also includes non-CSA members to compare if they also have a high need for information about their food.

## Conclusions

The aim of our study was to explore which factors influence trust in CSA and its farmers, and how strong the impact of these factors is when compared to each other. Our results suggest that trust in CSA and its farmers is high. Examining the factors that influence trust indicated that interaction and communication between producers and their members are important measures for trust-building. Reputation, supply of information, direct social interaction, the duration of CSA membership and attitudes toward short food chains are significant predictors of trustworthiness. Attitudes toward certification and the certification status of the farm itself did not impact trust. Therefore, it appears that in a CSA context, informal ways of demonstrating openness and credibility are more trusted than formal, institutionalized signals. The high importance of reputation and supply of information when compared to direct social interaction emphasized that trust can also be created in the absence of face-to-face interaction. This finding also suggests that the community in CSA is not only defined through face-to-face interaction but also generally through values such as openness and transparency. However, this also indicates that the creation of trust requires social skills that go far beyond farming.

Looking at the bigger picture, it is important to highlight that the solution to addressing decreasing trust in the dominant food system is not a complete replacement of conventional food supply with CSA farms. The co-existence of different approaches is necessary to cover different consumer needs and their demand for food. However, our study suggests the necessity of a transition toward a more regionalized and value-based food system. This would enable more farmers to at least partially adopt trust-creating elements from CSA and could help restore agriculture‘s integrity. Additionally, social media and new technologies such as apps, if customized for consumers’ needs, seem a promising way to enable continuous communication, knowledge exchange and complement traditional forms of creating trust. Overall, we conclude that the responsibility to provide transparency should not only lie with farmers. All actors involved in the food chain need to be more open and provide consumers with easily accessible, comprehensible, and relevant information about their food to increase trust in the food system and enable more sustainable consumption decisions.

## Supplementary Information

Below is the link to the electronic supplementary material.

Supplementary file1 (DOCX 36 kb)

## Abbreviations

AFN: Alternative food networks
CSA: Community-supported agriculture
