# Supplementary material for: Exploring member trust in German community-supported agriculture: a multiple regression analysis
Source: Agric Human Values. 2022 Nov 7;40(2):709–24. doi: 10.1007/s10460-022-10386-3 (PMC9638179; doi:10.1007/s10460-022-10386-3)
Supplement: Supplementary file 1 — Supplementary file1 (DOCX 36 kb) [file 10460_2022_10386_MOESM1_ESM.docx]

**Supplementary material**

**Annex 1: Survey**

**Part A**

First of all, I would like to ask some questions about your general shopping behavior and your fundamental attitude towards food.

| 1. **Please select five shopping criteria that are most important to you** |
| --- |
| □ Taste |
| □ Organic certification |
| □ Natural ingredients |
| □ Regional origin |
| □ Everyone in my household likes the product |
| □ Price |
| □ Freshness |
| □ Product is low in calories |
| □ Animal welfare |
| □ Seasonality of the product |
| □ Availability in my most frequented store |
| □ Quality |
| □ Long shelf life |
| □ Fair trade |
| □ Easy to prepare |
| □ Other, please state:_____________ |

1. **To what degree do you agree with the following statements?**

| **Statement** | **Fully agree** | **Rather agree** | **Neither agree nor disagree** | **Rather disagree** | **Fully Disagree** |
| --- | --- | --- | --- | --- | --- |
| I generally think a lot about what kind of food I purchase. | □ | □ | □ | □ | □ |
| I trust in governmental institutions responsible for controlling food safety (e.g. the Federal Office of Consumer Protection and Food Safety). | □ | □ | □ | □ | □ |
| I have a basic idea how the food that I mainly consume is produced. | □ | □ | □ | □ | □ |

| 1. **Where do you most frequently shop the food that you do not get from your CSA?** |
| --- |
| □ Discounter(e.g. Lidl, Aldi) |
| □ Supermarket(e.g. REWE, Edeka) |
| □ Organic supermarket(e.g. Bio Company, Denns) |
| □ Organic grocery store |
| □ Farmers’ market |
| □ Other, please state: |

| 1. **Please estimate the average share of organic food in per cent of your entire grocery shopping.** |
| --- |
| 0 % - 100 % |

**Part B**

In this part of the survey, I would like to ask some questions with regard to your CSA and the contact to your farmer and other members. In this part it is important that you answer all the questions because they are particularly important for the data evaluation.

| 1. **Which products do you obtain from your CSA?**   *Multiple answers are possible* |
| --- |
| □ Vegetables |
| □ Fruit |
| □ Meat |
| □ Juice |
| □ Bakery products |
| □ Eggs |
| □ Milk/ Dairy products |
| □ Other |

| 1. **How much money do you spend during the harvest season on food on top of the cost of your CSA harvest share per month.** (Please estimate the sum) |
| --- |
| ____ € |

| 1. **How much money do you spend off-harvest season on food on top of the cost of your CSA harvest share per month.** (Please estimate the sum) |
| --- |
| ____ € |

| 1. **What kind of agriculture does your CSA conduct?** |
| --- |
| □ My CSA applies organic principles but has no organic certification. |
| □ My CSA applies organic principles and has an organic certification. |
| □ My CSA applies conventional principles. |
| □ I am not sure. |

| 1. **What form of member participation does your CSA offer?** |
| --- |
| □ There is voluntarily work that members contribute to the CSA (e.g. farm/field days, helping to distribute the harvest products, help out in the office). |
| □ There is obligatory work that members contribute to the CSA (e.g. farm/field days, helping to distribute the harvest products, help out in the office). |
| □ I am not sure. |

| 1. **How satisfied are you with the degree of member participation** (e.g. farm/field days, helping to distribute the harvest products, help out in the office)**?** |
| --- |
| □ I would like to be able to participate more, namely in the following area (please state): |
| □ I would like to participate less, namely in the following area (please state): |
| □ The degree of participation is just right. |
| □ No comment. |

| 1. **For how long have you been a CSA member?** |
| --- |
| □ Less than a month |
| □ Between one month and less than three months |
| □ Between three months and less than six months |
| □ Between six months and less than a year |
| □ Between one year and less than 1.5 years |
| □ Between 1.5 years and less than two years |
| □ Between two years and less than 2.5 years |
| □ Between 2.5 years and less than three years |
| □ Between three years and less than four years |
| □ Between four years and less than five years |
| □ Between five years and less than six year |

1. **To what degree do you agree with the following statements?**

| **Statement** | **Fully agree** | **Rather agree** | **Neither agree nor disagree** | **Rather disagree** | **Fully Disagree** |
| --- | --- | --- | --- | --- | --- |
| The quality of my CSA produce meets my expectations. | □ | □ | □ | □ | □ |
| The price of the harvest share / products is appropriate. | □ | □ | □ | □ | □ |
| Due to my prior experience with the CSA, I think that the CSA will also meet my expectations regarding the quality of the products in the future. | □ | □ | □ | □ | □ |
| My harvest share has been delivered / has been available to me reliably | □ | □ | □ | □ | □ |
| Due to my prior experience with my CSA, I think that my harvest share will be delivered / will be available to me reliably in the future. | □ | □ | □ | □ | □ |
| Regarding food, my CSA and I share the same values. | □ | □ | □ | □ | □ |
| I would recommend to people who are important to me to join my CSA. | □ | □ | □ | □ | □ |
| I am willing to accept flaws caused by my CSA because I think that the operators generally do their best to avoid mistakes. | □ | □ | □ | □ | □ |

1. **To what degree do you agree with the following statements?**

| **Statement** | **Fully agree** | **Rather agree** | **Neither agree nor disagree** | **Rather disagree** | **Fully Disagree** |
| --- | --- | --- | --- | --- | --- |
| Transparent production processes are generally important to me. | □ | □ | □ | □ | □ |
| My CSA provides sufficient transparency regarding production and processing of their products. | □ | □ | □ | □ | □ |
| Being a member in a CSA increased my knowledge about food. | □ | □ | □ | □ | □ |
| It is important to me that information on my CSA’s food production are available online. | □ | □ | □ | □ | □ |
| I am satisfied with the amount of information that my CSA provides online. | □ | □ | □ | □ | □ |
| The content of information provided online by my CSA is interesting to me. | □ | □ | □ | □ | □ |
| It is important to me to know what my financial contribution is used for by my CSA. | □ | □ | □ | □ | □ |
| My CSA provides sufficient information on what the members’ financial contributions are used for. | □ | □ | □ | □ | □ |
| I assume that the information provided by my CSA are correct. | □ | □ | □ | □ | □ |

1. **To what degree do you agree with the following statements?**

| **Statement** | **Fully agree** | **Rather agree** | **Neither agree nor disagree** | **Rather disagree** | **Fully Disagree** |
| --- | --- | --- | --- | --- | --- |
| It is important to me to know the personal background of the farmer and to know what their motivation to grow food is. | □ | □ | □ | □ | □ |
| The possibility to directly interact with farmers increases my faith that my CSA farmer(s) conduct agriculture responsibly. | □ | □ | □ | □ | □ |
| There is a sense of community between members and farmer(s) in my CSA. | □ | □ | □ | □ | □ |
| It is important to me to have the opportunity to participate in my CSA. | □ | □ | □ | □ | □ |
| In my CSA there are sufficient opportunities for member participation. | □ | □ | □ | □ | □ |
| Interactions with other CSA members are important to me. | □ | □ | □ | □ | □ |
| In my CSA I have sufficient interactions with other members. | □ | □ | □ | □ | □ |
| It is important to me to be able to get direct feedback from farmer(s) in case I have questions regarding their products or production methods. | □ | □ | □ | □ | □ |
| In my CSA I get sufficient feedback regarding products and production methods. | □ | □ | □ | □ | □ |
| The social interactions in my CSA are an enrichment for me. | □ | □ | □ | □ | □ |
| It is important to me to have the possibility to visit the CSA to personally get an idea of the production of my food. | □ | □ | □ | □ | □ |
| I expect my CSA to regularly ask me for my satisfaction as a member. | □ | □ | □ | □ | □ |

1. **To what degree do you agree with the following statements?**

| **Statement** | **Fully agree** | **Rather agree** | **Neither agree nor disagree** | **Rather disagree** | **Fully Disagree** |
| --- | --- | --- | --- | --- | --- |
| Organic certification is generally an important criterion to me when shopping groceries. | □ | □ | □ | □ | □ |
| Organic certification gives me security that the product has attributes that I cannot observe. | □ | □ | □ | □ | □ |
| I know the different standards that different organic labels entail. | □ | □ | □ | □ | □ |
| When I buy organic food, I pay attention which specific organic label the product has. | □ | □ | □ | □ | □ |
| I know where I can get information on organic labels. | □ | □ | □ | □ | □ |
| If food has an organic label, I do not need additional information on the product. | □ | □ | □ | □ | □ |
| Organic certification ensures that farmers produce food according to the required standard. | □ | □ | □ | □ | □ |
| Organic food production is of high importance for my CSA (regardless whether they have organic certification or not). | □ | □ | □ | □ | □ |
| It does not matter to me whether the products of my CSA have organic certification or not. | □ | □ | □ | □ | □ |

1. **To what degree do you agree with the following statements?**

| **Statement** | **Fully agree** | **Rather agree** | **Neither agree nor disagree** | **Rather disagree** | **Fully Disagree** |
| --- | --- | --- | --- | --- | --- |
| I generally trust in my CSA. | □ | □ | □ | □ | □ |
| I generally trust in the farmer(s) of my CSA. | □ | □ | □ | □ | □ |
| I trust that my CSA tries to achieve the best possible product quality. | □ | □ | □ | □ | □ |
| I trust that my CSA does not overcharge for their products. | □ | □ | □ | □ | □ |
| I trust that my CSA provides their employees with fair labor conditions. | □ | □ | □ | □ | □ |
| I trust that my CSA sticks to environmental standards. | □ | □ | □ | □ | □ |
| I trust that my CSA can guarantee for the safety of the food that they supply. | □ | □ | □ | □ | □ |
| Short supply chains are more reliable than long supply chains. | □ | □ | □ | □ | □ |
| In times of crises, it is important to me not to be reliable on long supply chains. | □ | □ | □ | □ | □ |

**Sociodemographic Data**

Finally, I have some questions regarding your person.

| 1. **What is your gender?** | | | |
| --- | --- | --- | --- |
| □ female | □ male | □ diverse | □ No comment |

| 1. **Please indicate your age.** | |
| --- | --- |
| □ 18-29 | □ 30-44 |
| □ 45-59 | □ 60-65 |
| □ Above 65 | □ No comment |

| 1. **Please indicate your highest educational attainment.** | |
| --- | --- |
| □ University degree | □ Intermediate school (Mittlere Reife) |
| □ Advanced technical college certificate | □ Secondary education (Abitur/Hochschulreife) |
| □ Completed vocational training | □ University college degree |
| □ PhD | □ other, namely: |
| □ No comment |  |

| 1. **Do you live in an urban or a rural area?** | | |
| --- | --- | --- |
| □ urban | □ rural | □ no comment |

| 1. **Please tell me the first three digits of your residence’s zip code.** |
| --- |
| ______ |
| □ No comment |

| 1. **Please indicate the average monthly gross income of your household. (Only consider persons that you are sharing the harvest share with, please)** |
| --- |
| □ Less than 500 Euro |
| □ 500 up to less than 1,000 Euro |
| □ 1,000 up to less than 1,500 Euro |
| □ 1,500 up to less than 2,000 Euro |
| □ 2,000 up to less than 2,500 Euro |
| □ 2,500 up to less than 3,000 Euro |
| □ 3,000 up to less than 3,500 Euro |
| □ 3,500 up to less than 4,000 Euro |
| □ 4,000 up to less than 4,500 Euro |
| □ 4,500 up to less than 5,000 Euro |
| □ 5,000 up to less than 5,500 Euro |
| □ 5,500 up to less than 6,000 Euro |
| □ More than 6,000 Euro |
| □ No comment |

| 1. **How many persons contribute to the household income including yourself? (Only consider persons that you are sharing the harvest share with, please)** |
| --- |
| ___ |
| □ No comment |

| 1. **How many people live in your household? (Only consider persons that you are sharing the harvest share with, please)** |
| --- |
| Number: ___ |
| □ No comment |

| 1. **Please use the free text field for further comments on the survey or on the topic trust in food.** |
| --- |
|  |

**Annex 2: Table S1. Factor loadings and Cronbach’s alpha for variable scales.** Each variable represents the factor loadings from a separate exploratory factor analysis.

| **Scale** | **Item** | **Factor Loading** | **Cronbach’s Alpha** |
| --- | --- | --- | --- |
| Reputation | The quality of my CSA produce meets my expectations. | 0.694 | 0.84 |
|  | The price of the harvest share / products is appropriate. | 0.649 |  |
|  | Due to my prior experience with the CSA, I think that the CSA will also meet my expectations regarding the quality of the products in the future. | 0.792 |  |
|  | My harvest share has been delivered / has been available to me reliably | 0.514 |  |
|  | Due to my prior experience with my CSA, I think that my harvest share will be delivered / will be available to me reliably in the future. | 0.670 |  |
|  | Regarding food, my CSA and I share the same values. | 0.514 |  |
|  | I would recommend to people who are important to me to join my CSA. | 0.654 |  |
|  | I am willing to accept flaws caused by my CSA because I think that the operators generally do their best to avoid mistakes. | 0.587 |  |
| Supply of information | Transparent production processes are generally important to me. | 0.395 | 0.74 |
|  | My CSA provides sufficient transparency regarding production and processing of their products. | 0.699 |  |
|  | Being a member in a CSA increased my knowledge about food. | 0.397 |  |
|  | I am satisfied with the amount of information that my CSA provides online. | 0.655 |  |
|  | The content of information provided online by my CSA is interesting to me. | 0.573 |  |
|  | I assume that the information provided by my CSA are correct. | 0.649 |  |
|  | My CSA provides sufficient information on what the members’ financial contributions are used for. | 0.607 |  |
| Social interaction | It is important to me to know the personal background of the farmer and to know what their motivation to grow food is. | 0.530 | 0.84 |
|  | The possibility to directly interact with farmers increases my faith that my CSA farmer(s) conduct agriculture responsibly. | 0.524 |  |
|  | There is a sense of community between members and farmer(s) in my CSA. | 0.576 |  |
|  | It is important to me to have the opportunity to participate in my CSA. | 0.684 |  |
|  | In my CSA there are sufficient opportunities for member participation. | 0.422 |  |
|  | Interactions with other CSA members are important to me. | 0.697 |  |
|  | In my CSA I have sufficient interactions with other members. | 0.401 |  |
|  | It is important to me to be able to get direct feedback from farmer(s) in case I have questions regarding their products or production methods. | 0.536 |  |
|  | In my CSA I get sufficient feedback regarding products and production methods. | 0.503 |  |
|  | The social interactions in my CSA are an enrichment for me. | 0.719 |  |
|  | It is important to me to have the possibility to visit the CSA to personally get an idea of the production of my food. | 0.592 |  |
| Organic certification | Organic certification is generally an important criterion to me when shopping groceries. | 0.646 | 0.78 |
|  | Organic certification gives me security that the product has attributes that I cannot observe. | 0.655 |  |
|  | I know the different standards that different organic labels entail. | 0.687 |  |
|  | When I buy organic food I pay attention which specific organic label the product has. | 0.638 |  |
|  | I know where I can get information on organic labels. | 0.587 |  |
|  | If food has an organic label I do not need additional information on the product. | 0.370 |  |
|  | Organic certification ensures that farmers produce food according to the required standard. | 0.528 |  |
| Trust | I generally trust in my CSA. | 0.717 | 0.84 |
|  | I generally trust in the farmer(s) of my CSA. | 0.727 |  |
|  | I trust that my CSA tries to achieve the best possible product quality. | 0.711 |  |
|  | I trust that my CSA does not overcharge for their products. | 0.721 |  |
|  | I trust that my CSA provides their employees with fair labor conditions. | 0.580 |  |
|  | I trust that my CSA sticks to environmental standards. | 0.675 |  |
|  | I trust that my CSA can guarantee for the safety of the food that they supply. | 0.527 |  |
